# Pure phase-locking of beta/gamma oscillation contributes to the N30 frontal component of somatosensory evoked potentials

**DOI:** 10.1186/1471-2202-8-75

**Published:** 2007-09-18

**Authors:** Guy Cheron, Ana Maria Cebolla, Caty De Saedeleer, Ana Bengoetxea, Françoise Leurs, Axelle Leroy, Bernard Dan

**Affiliations:** 1 Laboratory of Neurophysiology and Movement Biomechanics, Université Libre de Bruxelles (ULB), CP 168, 50 Av F Roosevelt, Brussels, Belgium; 2 Laboratory of Electrophysiology, Université de Mons-Hainaut, Belgium; 3 Department of Neurology, Hopital Universitaire des Enfants Reine Fabiola, Université Libre de Bruxelles (ULB), Belgium

## Abstract

**Background:**

Evoked potentials have been proposed to result from phase-locking of electroencephalographic (EEG) activities within specific frequency bands. However, the respective contribution of phasic activity and phase resetting of ongoing EEG oscillation remains largely debated. We here applied the EEGlab procedure in order to quantify the contribution of electroencephalographic oscillation in the generation of the frontal N30 component of the somatosensory evoked potentials (SEP) triggered by median nerve electrical stimulation at the wrist. Power spectrum and intertrial coherence analysis were performed on EEG recordings in relation to median nerve stimulation.

**Results:**

The frontal N30 component was accompanied by a significant phase-locking of beta/gamma oscillation (25–35 Hz) and to a lesser extent of 80 Hz oscillation.

After the selection in each subject of the trials for which the power spectrum amplitude remained unchanged, we found pure phase-locking of beta/gamma oscillation (25–35 Hz) peaking about 30 ms after the stimulation. Transition across trials from uniform to normal phase distribution revealed temporal phase reorganization of ongoing 30 Hz EEG oscillations in relation to stimulation. In a proportion of trials, this phase-locking was accompanied by a spectral power increase peaking in the 30 Hz frequency band. This corresponds to the complex situation of 'phase-locking with enhancement' in which the distinction between the contribution of phasic neural event versus EEG phase resetting is hazardous.

**Conclusion:**

The identification of a pure phase-locking in a large proportion of the SEP trials reinforces the contribution of the oscillatory model for the physiological correlates of the frontal N30. This may imply that ongoing EEG rhythms, such as beta/gamma oscillation, are involved in somatosensory information processing.

## Background

In the classical view (evoked or additive model), evoked potentials reflect a sequential 'bottom-up' processing of sensory stimulus inducing specific sequence of monophasic 'evoked' potential peaks that are embedded in 'random background' electroencephalogram (EEG). They are considered as distinctive components with fixed latency and polarity, reflecting anatomically distinct generators whose activity is independent from the spontaneous EEG, which is considered as noise that must be ruled out by means of averaging [1-3]. In this conceptual view, the EEG phase distribution is unaffected by the stimulation and the amplitude reduction of an evoked potential component is interpreted independently of temporal reorganization of the ongoing EEG.

An alternative view (oscillation model) suggested by pioneer experiments [4,5] pointed out the fact that evoked potentials might result from phase-locking or phase-reset of the basic EEG rhythms within specific frequency bands, as a response to external stimulation [6]. Evidence of stimulus-induced phase-locking has been reported by several groups, using a variety of signal analysis methods [7-12].

In this context, the concept of synchronized resonances has been introduced by Basar in 1980 [6]. In accordance to the general theory of resonance phenomena it was proposed that a sensory stimulation gives rise to 'evoked' or 'induced' EEG rhythms in several frequency bands. The 'evoked' rhythms are phase-locked to the stimulus and can be observed in the averaged evoked potentials, while the 'induced' rhythms are cancelled out during averaging because of the jitter in the latency from one trial to the next [13]. Contribution of the oscillatory model has been demonstrated in the generation of visual and auditory evoked potentials [4-7,11].

Here we tested whether this model can apply in somatosensory evoked potentials (SEP). We focused on the frontal N30 component of SEP, as it is highly sensitive to interference or gating from concomitant involvement of the brain in sensory, motor and mental activities [14-19]. This wave is specifically modulated by electrical stimulation of the internal part of the globus pallidus or the subthalamus nuclei of Parkinsonian patients [20], suggesting that it may represent a reliable physiological index of the dopaminergic motor pathways [21]. Investigation of the frontal N30 component has increasingly been used in a host of clinical conditions [22-26]. However, the physiological interpretation and the origin of the frontal N30 are still debated [21,27,28].

The aim of this work was to study whether reorganization of background EEG activity contributes to the generation of the N30 component or whether this component essentially results from the activity of a generator unrelated to ongoing EEG rhythms, as posited in the additive model. Confirmation of the latter hypothesis would imply that future research should continue to concentrate on the characterization of discrete local generators through improved cancelling out of ongoing EEG rhythms. In this view, inversion of the polarity of the N30 component, as illustrated in patients with early acquired basal ganglia lesions [25,26] might be interpreted as reflecting a sign switch in cortical synaptic currents. In contrast, if oscillatory phase-resetting contributes to the N30, future analyses should specifically address the relationship between stimulation and the dynamic organization of background EEG, including phase-synchronization of ongoing rhythms across various spatiotemporal scales. In the above example, N30 polarity inversion would reflect abnormal phase resetting of ongoing EEG rhythms rather than synaptic changes. This might also provide new insights into the mechanisms underlying the facilitation of information transfer and in particular perceptual binding [29-31].

Making progress in the debate between the additive and the oscillatory models [32-34] has become crucial because evoked potentials are increasingly used in clinic as physiological and neuropsychological index of brain areas or as link with other functional approaches such as fMRI and the underlying network dynamics. It must be borne in mind, however, that the two models are not mutually exclusive. For example, phase-locked components (transitory or oscillatory) may be present in both the additive and oscillatory models. Nevertheless, it was shown that phase-locking and power enhancement of theta, alpha and gamma rhythms may evolve independently in aging and development [35-39], indicating the existence of different physiological mechanisms.

The approach of time-frequency analysis to single EEG trials we used was developed by Makeig et al. (2002) [11]. This method allows to identify a superimposed neural contribution in the latency range of the evoked response by computing changes in the power spectrum in comparison with the pre-event activity. Moreover, it allows to detect phase reorganization of EEG rhythms. However, a limitation of this approach was underlined in a recent simulation study demonstrating that the addition of a phasic signal on the ongoing EEG was able to induce a phase resetting in some EEG frequency bands [40]. Indeed, there are two variants of the oscillation hypothesis. The first and simple situation is the *pure phase resetting *during which the occurrence of an event leads to resetting the phase of ongoing EEG rhythms without any change in the amplitude modulation of the EEG. This unequivocal situation has been described for the N1 component of the auditory [10,5] and visual evoked potentials [34,9]. The second, more complex situation is described as *phase resetting with enhancement *during which the sensory stimulation induces an increase in EEG amplitude in addition to phase resetting [6]. In this case, it is not possible to distinguish the activity generated by phasic neural events (independent of spontaneous EEG) from those linked to EEG phase resetting [40]. This is the reason why we investigated whether phase-locking and event-related power spectral perturbation in specific EEG frequency bands occur in the production of this component. We focused our analysis on the presence or absence in the different trials of a pure phase-resetting participating in the production of the N30 SEP component, as pure phase-resetting (i.e. without any power enhancement) would demonstrate the contribution of the oscillatory model to N30 generation.

## Results

### Grand averaged analysis

**Figure 1:**
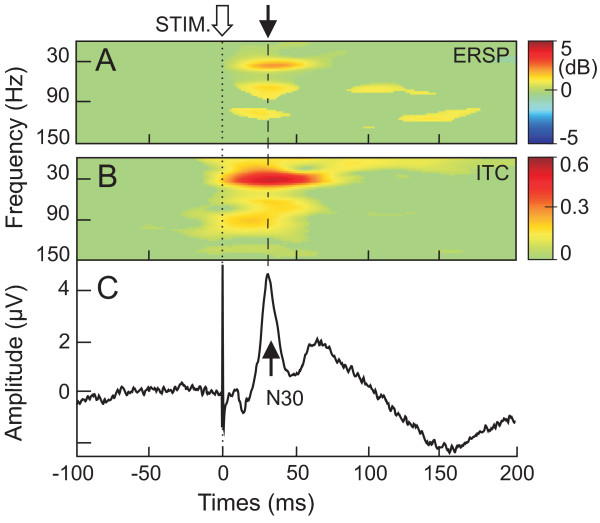
**Grand average analysis of N30 component**. **A **and **B **Grand average of time/frequency template from ERSP and ITC analysis, respectively. **C**, concomitant grand averaging of the frontal N30 component recorded from F4 during median nerve stimulation at the wrist. Note that the peak of ERSP and ITC value in the beta range (25–35 Hz) coincided with the N30 latency peak. Colored areas show ERSP and ITC that are statistically significant (p < 0.001).

Figure 1 shows grand average of the SEPs (Fig. 1C) together with the corresponding ERSP (Fig. 1A) and ITC (Fig. 1B) presentations (3000 trials, n = 7 subjects). The major notable phenomena are the phase-locking and amplitude (power) enhancement in the post-stimulus period, corresponding to the expression of the N30 in the averaged SEP (Fig. 1C). The phase-locking in the beta/gamma band oscillation (mean frequency of 33.1 ± 1.3 Hz) peaked at a latency of 34.9 ± 5.2 ms and was quantified by an ITC value reaching 0.53 ± 0.17. At this latency, the beta/gamma cluster presented an ITC value greater than 0.3 extending from 20.4 ± 2.9 to 42.1 ± 4.4 Hz, respectively. An ERSP cluster occurred at the same latency than this ITC cluster (peaking at 35.2 ± 6.3 ms) reaching a maximal value of 2.4 ± 1.5 dB. Smaller ITC values were measured for faster and slower rhythms than 30 Hz.

**Figure 2:**
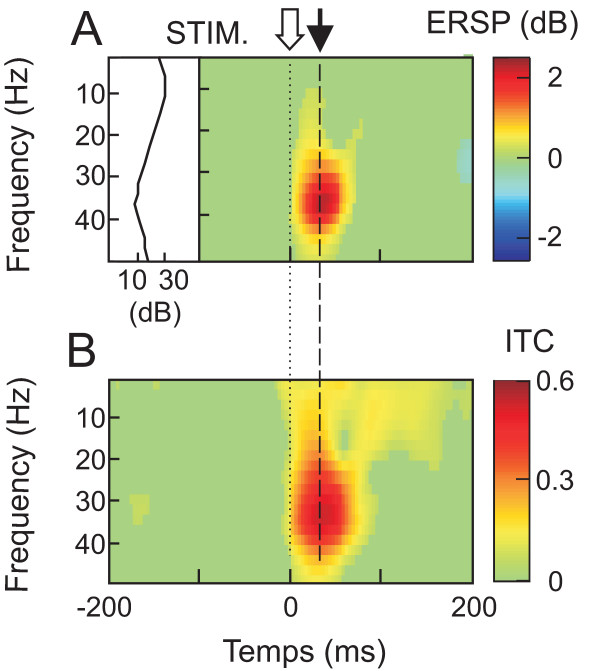
**Grand average of time/frequency template focused on alpha-beta/gamma frequencies**. **A**, ERSP analysis and mean power spectrum in the pre-stimulus period. **B**, ITC analysis. Colored areas show ERSP and ITC that are statistically significant (p < 0.001). The analysis originated from the same set of data as in Figure 1.

For alpha rhythm (mean frequency of 13.2 ± 2.8 Hz), a diffuse band of ITC value reaching the maximum (0.29 ± 0.10) at the latency of 54.8 ± 40.5 ms was present but no significant ERSP value (maximal value of 0.83 ± 0.17 dB) was found at this frequency (Fig. 2).

For faster gamma rhythm, a cluster with a diffuse shape was obtained (mean ITC of 0.37 ± 0.16, dark yellow in Fig. 1B). The maximal gamma ITC corresponded to a frequency peak of 80.1 ± 23.0 Hz occurring at 23.8 ± 10.2 ms. The grand average ERSP map showed two distinct clusters around 70 Hz and 110 Hz, respectively (yellow clouds in Fig. 1A). However, in contrast to the 30 Hz ERSP cluster, these higher frequency rhythms were differently expressed by the subjects and mainly provided by two of them.

### Identification of pure phase-locking

Identification of phase resetting of spontaneous EEG activities in certain frequency bands requires the demonstration of the presence of these EEG oscillations in the absence of stimulus. We therefore measured the power spectrum of the spontaneous 30 Hz oscillation in each single trial excluding the periods of evoked activity. For all the recorded trials, the mean of the 30 Hz power spectrum represented a value of 17.1 ± 4.9 dB. This suggests that the 30 Hz oscillation, may be involved in a phase-locking process. For indication, the 30 Hz power spectrum represented ~65% of the power spectrum of the dominant alpha-mu rhythm (~10 Hz) measured during the same period (26.4 ± 5.3 dB).

As it was demonstrated that in case of *phase locking with enhancement*, which corresponds to the present situation, it was impossible to distinguish the possible contribution of EEG phase-resetting from phasic activity [40], we focused our analysis on the trials of individual subjects in which the power spectrum in the beta/gamma band respected the criterion described in equation n°4 and thus remained unchanged after stimulation.

**Figure 3:**
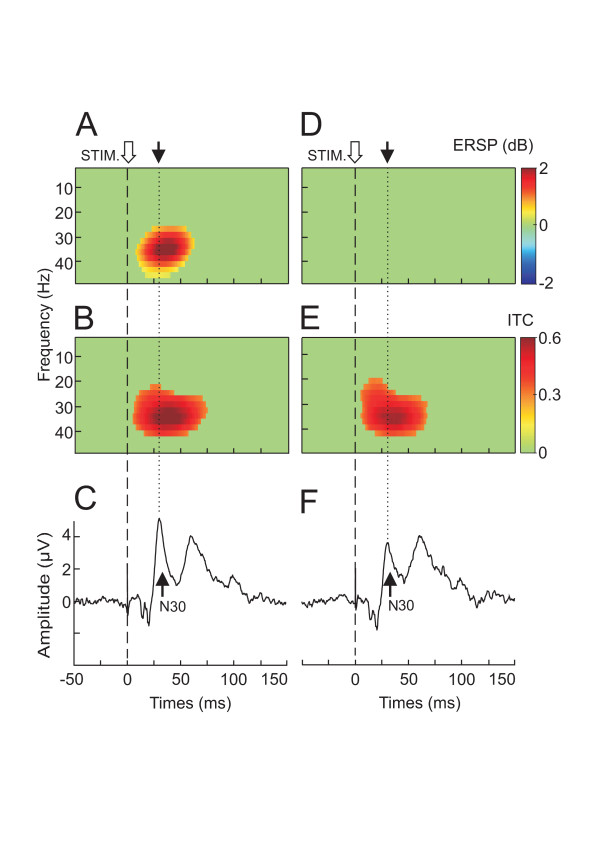
**Selection of pure phase-locking illustrated for one subject**. **A**,**B **and **C**, ERSP, ITC and N30 evoked potentials, respectively, recorded when all the trials were taken into account. Note that this situation corresponds to phase-locking with amplitude enhancement where the N30 component was accompanied by significant ERSP (A) and ITC (B) cluster in the beta frequency range. **D**, **E **and **F **ERSP, ITC and N30 evoked potentials, respectively recorded when only the trials for which no amplitude enhancement were taken into account. This situation corresponds to pure phase-locking where the N30 component was only accompanied by the ITC cluster in the beta range. Note the reduction of the N30 amplitude in the pure phase-locking situation (F). Colored areas show ERSP and ITC that are statistically significant (p < 0.001).

For each of the 7 subjects, it was possible to identify a large percentage of trials (62 ± 16%) for which the power spectrum remained unchanged within the whole frequency range spanning 1 to 50 Hz. Figure 3 illustrates the result of this selection in one subject. When all the trials were taken into account a clear ERSP cluster appeared in the beta/gamma band (Fig. 1A (grand average data) and Fig. 3A (single subject data)). This power increase was concomitantly accompanied by a significant ITC cluster (Fig. 1B (grand average data) and Fig. 3B (single subject data)). After the selection, although no more ERSP cluster could be found (Fig. 3D), a significant ITC cluster in the beta/gamma band was still present (Fig. 3E). The maximal ITC value, duration, peak latency and frequency band of the ITC cluster were not significantly changed by the trial selection (Table 1). Conversely, the N30 amplitude was significantly reduced by the selection (4.5 ± 1.7 μV versus 3.6 ± 1.2 μV, p < 0.02, Fig. 3C, F). In spite of this amplitude reduction, the N30 component conserved its initial morphology and peaked at the same latency (31.3 ± 0.9 ms versus 31.1 ± 0.9 ms). After the selection procedure the relative percentage of the 30 Hz power remained the same (66.6%).

**Table 1:** Characterization of ITC clusters

Subject	ITC max value		ITC duration (ms)		ITC max value latency (ms)		ITC frequency band (Hz)	
	All trials	trials with no ERSP change	All trials	trials with no ERSP change	All trials	trials with no ERSP change	All trials	trials with no ERSP change

1	0.51	0.55	69.3	60.8	42.0	41.4	19 – 44	23 – 44
2	0.36	0.38	35.5	41.5	37.7	34.6	26 – 38	25 – 38
3	0.63	0.58	70.3	61.3	34.2	31.7	21 – 42	18 – 42
4	0.57	0.60	55.2	51.4	28.9	30.2	21 – 38	19 – 38
5	0.36	0.37	44.9	49.4	28.0	28.7	21 – 38	23 – 38
6	0.43	0.43	50.2	53.7	34.6	32.1	18 – 49	17 – 49
7	0.85	0.84	76.8	75.1	39.0	38.3	17 – 46	20 – 46

mean	0.53	0.54	57.4	56.1	34.9	33.8	20 – 42	21 – 42
SD	0.17	0.16	15.1	10.8	5.2	4.56	3 – 4	3 – 4

**These ITC parameters were collected from EEGLab template**. One-way ANOVA test shows that for each of the four presented parameters, the values measured for all the trials versus those measured for the trials with no ERSP change were not significantly different.

Although we selected trials for which power variation was statistically comparable to power distribution in the stimulus-free period, we compared the results obtained for the trials for which beta/gamma oscillation power was between RMSfree and RMSfree-1SD (1^st ^group) and the one for which beta/gamma power was between RMSfree and RMSfree+1SD (2^nd ^group). No significant differences between the two groups of trials in the ITC value (0.49 ± 0.13 versus 0.61 ± 0.13; p = 0.7) and N30 amplitude (3.1 ± 0.9 μV versus 4.2 ± 1.6 μV; p = 0.6) were found. This indicates that both significant phase-locking and N30 component were already present if only the trials presenting a power decrease were taken into account.

### Phase-locking analysis

**Figure 4:**
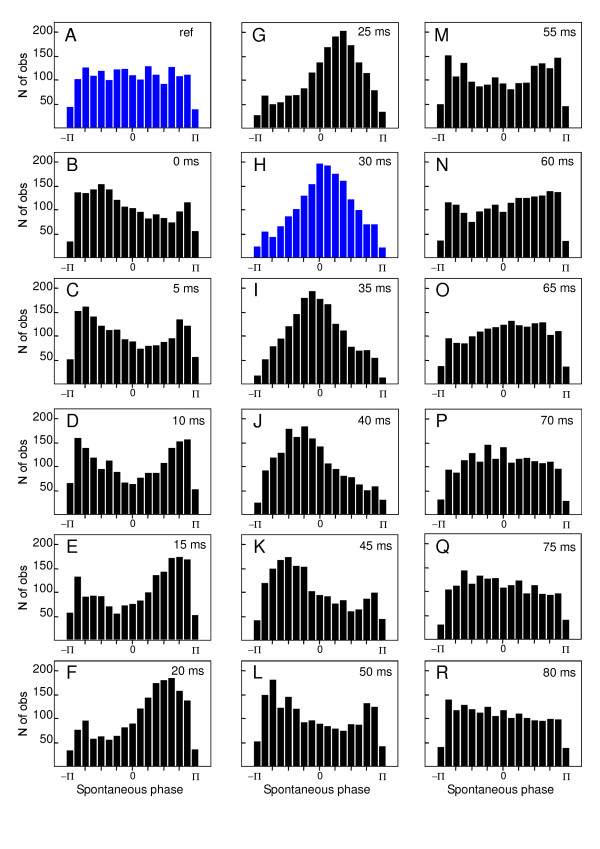
**Reorganization of the spontaneous phase of beta oscillation**. Cumulative histograms of the spontaneous phase of single trials beta band oscillation (25–35 Hz) recorded in all the subjects (pooled as a single set) and selected with respect to the pure phase-locking criteria. Horizontal axis ranges from -π to π. Vertical axis corresponds to the number of trials. **A**, histogram of the pre-stimulus reference period ([-60 ms,-5 ms]). **B-R**, succession of histograms calculated every 5 ms, from 0 to 80 ms with respect to the stimulation time. Note the progressive reorganization of phase distribution peaked at 0 radian at 30 ms.

In order to demonstrate the temporal reorganization of beta/gamma oscillations following the stimulation, the instantaneous phase of each selected trial was calculated for each subject. Figure 4 shows the spontaneous phases of all selected trials of all the subjects (n = 1739) in a cumulative histogram. Before the stimulus, the histogram of phase distribution corresponds to uniform density function (Fig. 4A). After the stimulus a phase alignment occurred and the phase distribution became gradually more peaked.

**Figure 5:**
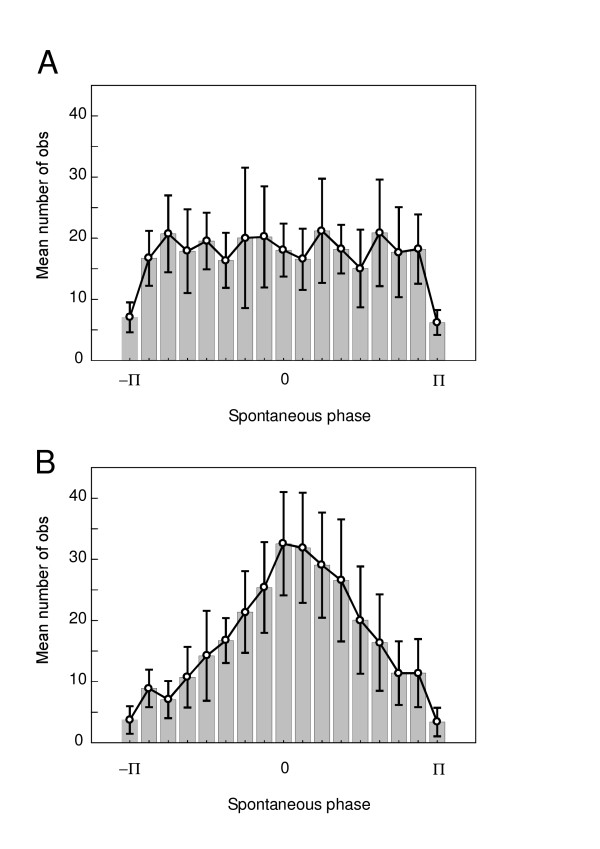
**Mean histogram of the spontaneous phase of beta oscillation**. Mean and SD of phase histograms (same data as in Fig. 3, but calculated here on each individual subject). **A**, histogram before stimulus (at -60 ms). **B**, histogram after stimulus (at 30 ms).

**Figure 6:**
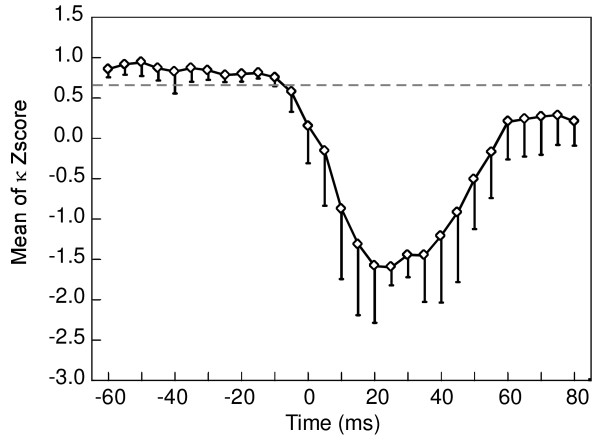
**Statistical analysis of the beta phase distribution**. Evolution of the Z score (mean and SD) of *the Kuiper's statistic κ*. Z score is significant (p < 0.05) below a value of 0.68. Note that the highest significant values are reached around a post-stimulus time of 30 ms.

The peak of the distribution reached a phase value of 0 radian at the latency of the N30 component (Fig. 4H). The comparison between the mean histogram calculated for the -60 ms pre-stimulus time (Fig. 5A) and the 30 ms post-stimulus time (Fig. 5B) showed a clear distinction between uniform and peaked distribution at 0 radian. The conservation of a same range of standard deviation throughout the distribution provided evidence for the reliability of the effect across subjects. Figure 6 illustrates the Z score of the Kuiper's statistic *κ*. The difference in phase distribution between the pre-stimulus reference period ([-60 ms,-5 ms]) and the post-stimulus times was significant (p < 0.05) below a Z score value of 0.68. This level was reached for each analyzed time after the stimulus and became highly significant around the N30 latency, reaching a value of -1.44 ± 0.28.

## Discussion

We found that the frontal N30 component of the SEP was characterized by a significant increase of the power spectrum of beta/gamma rhythm peaking around 30 Hz. This event-related spectral perturbation was accompanied by significant phase-locking of beta/gamma oscillation peaking at the latency of the frontal N30 component. In terms of the oscillation model of evoked potentials this situation corresponds to *phase resetting with enhancement*. In this situation, it is difficult to distinguish between the contribution of phasic neuronal activation versus phase resetting of ongoing EEG oscillation [40]. However, we demonstrated that for each subject a significant percentage of EEG trials show a beta/gamma phase-locking in the absence of any increase of the EEG power at the latency of the N30 component. This *pure phase resetting *indicates that some of the frequency content of the ongoing EEG (beta/gamma range) was phase-reset by the sensory stimulation and contributed to the frontal N30 amplitude.

We have focused the present paper on this beta/gamma oscillation because the ITC and ERSP values were maximal for this frequency band and that they coincide with the N30 latency peak. However, the absence of alpha power enhancement in the present data does not exclude the influence of its partial phase-locking on the evoked potentials amplitude.

It is generally admitted that phase-resetting implies 3 main requirements [41]: (1) oscillation at the dominant frequency of the evoked response must be present in the pre-stimulus period; (2) the transition between the pre- to the post-stimulus periods must involve phase concentration; (3) this phase transition must occur without concomitant increase in power at the dominant frequency of the ERP [40]. The present findings respected these 3 criteria. With respect to the first criterion, power spectrum analysis in the pre-stimulus period revealed that spontaneous beta/gamma oscillations are present and that they might thus be involved in phase-locking process. This criterion is reminiscent of the oscillatory susceptibility rule of Basar (1992)[42].

In the same line of evidence, this necessary condition for a plausible physiological contribution of beta/gamma oscillation phase-locking to the N30 component is reinforced by previous demonstration of spontaneous cortical activities in this frequency band in the sensori-motor areas. In humans, beta rhythm has been recorded by intra cerebral recordings in the pre- and post-central gyrus and in the frontal medial cortex [43]. In monkeys, field-potential oscillations in the 20–30 Hz range have been reported [44] and were supported by synchronous oscillatory activity in a large number of cortical neurons [45] providing synchronisation of neuronal firing between somatosensory and motor areas [46].

As time frequency measures have revealed the existence of phase reset with power enhancement of beta/gamma oscillation, we singled out the trials for which such enhancement did not occur in order to analyze our findings in the framework of the second criteria. These selected trials represented about 62% of the original trials. In this way, we also complied with the third criteria of a strict absence of power enhancement in these selected trials. We demonstrated that the stimulus induced a temporal reorganization of the spontaneous phase of the ongoing beta/gamma oscillation in these trials. The absence of a concomitant power increase in these trials rules out the possibility that an evoked *de novo *beta/gamma rhythm might explain the phase transition of the ongoing beta/gamma oscillation. The presence of significant ITC value and N30 amplitude when only the trials for which the power of beta/gamma oscillation decreases after the stimulation reinforces the contribution of ongoing EEG phase modulation in the N30 generation. However, it is important to emphasize that the presence of this pure phase locking does not exclude that evoked phasic activity occurs when all the trials were taken into account. Indeed, when the trials presenting power enhancement were rejected, we found that the N30 amplitude was significantly decreased. As ITC values remained the same, this amplitude reduction indicates the contribution of phasic activation to the N30 amplitude. This result was in line with theoretical [47] and experimental [48] studies demonstrating that phase and amplitude modulation could participate together in evoked potentials generation.

It was previously shown that median nerve stimulation was able to trigger an increase in the power of the 10 and 20 Hz oscillations [49], but only after a delay of 300 ms, which is 10 times later than the occurrence of the present 30 Hz oscillation. The same type of stimulation evoked an increase in the power of beta/gamma oscillation peaking around 500 ms with a latency onset of 300 ms [50]. These authors showed a decrease in beta oscillation during hand manipulation whereas the amplitude of P140/N200 SEP components was increased. However, the behaviour of early SEP components was not the focus of these studies.

Event-related synchronisation of gamma rhythms (40–60 Hz) to hand movement onset and offset [43] may correspond to the present clusters of gamma ITC recorded during median nerve stimulation. Indeed, the present paradigm produced small twitches of the thumb, inducing an afferent sensory feedback. However, the peak latency of the gamma phase-locking occurred too early (~25 ms) to originate from thumb movement feedback.

Resonance in the basal ganglionic-thalamo-cortical loop could be implicated in gamma oscillation triggered by sensory stimulation. The pallidum and the subthalamic nucleus form a functional network that resonates at 70 Hz in the presence of a normal dopaminergic drive [51]. This rhythm is replaced by slower oscillations in the off-state of Parkinsonian patients [51], which could be related to the N30 alteration in the Parkinsonian off-state [52,22]. Recently, it was suggested that the 30 Hz oscillation in the subthalamic nucleus that is suppressed during finger actual movement or mental imagery in Parkinsonian patients could be physiological and present in normal subjects [53].

The presence of a *pure phase resetting *in a large percentage of the SEP trials is the key element of the present study. It resembles the situation of the N1 components evoked by visual [11,34] or auditory stimuli [12]. The transition of the spontaneous phase from a pre-stimulus uniform distribution to a peaked form at 0 radian at the time of the N30 component demonstrates the reorganization of the ongoing beta/gamma rhythm. As proposed in the case of many ERP components [6] in addition to this phase resetting effect, the rest of the evoked component may be generated by coherent phasic activation of pyramidal neurons via thalamo-cortical input, as predicted by the classical view.

The present result could be integrated in the concept of synchronized resonances [6]. As described for the auditory 40 Hz oscillation [54], the N30 related beta/gamma rhythm may be viewed as a more global mechanism working in parallel to the stimuli processing of the somatosensory pathway. The phase-locking of this rhythm allows the placement of the sensory signal in the temporal context taking into account the intrinsic functional state of the brain at the arrival time of the stimulus.

The fact that we found power increase in some trials and not in others corroborates an integrative view that the classical and the oscillation models are not necessarily in opposition, but that their respective contribution must be clarified before tempting physiological or clinical conclusions.

As the activity recorded at one scalp channel sums activity from several cortical source areas [11], the question of the origin of the phase-locked and non-phase-locked activities cannot be fully addressed without using multiple channels recording.

Phase resetting of both local field potential and single-unit activity representative of the ongoing motor cortical beta (15–30 Hz) rhythms has been demonstrated in pyramidal tract stimulation in monkeys [55]. This view is also supported by in vitro and in vivo physiological studies [56] showing enhanced oscillation when neurons fire in-phase with the oscillation field. This could explain at a cellular level how the depolarisation induced by the sensory stimulus is able to reset the oscillating phase and bring the system into a synchronous attractor basin at the latency of the N30 component.

## Conclusion

The present study demonstrates that the frontal N30 component of the SEP is characterized by an increase of the power spectrum of beta/gamma rhythm peaking at 30 Hz and by a concomitant increase of the phase-locking. The fact that we found a pure phase-locking (without power enhancement) in about two thirds of the trials accompanied by a reorganization of the spontaneous phase of the ongoing beta/gamma rhythm constitute evidence for the contribution of the oscillation model to the production of the frontal N30 component. The concomitant increase of the beta/gamma power in about one third of the trials indicating the contribution of phasic signal (additive model) implies interaction between the physiological mechanisms of stimulus phasic related component and phase-resetting of ongoing spontaneous oscillations.

## Methods

### Subjects and conditions

The data were collected from 7 normal volunteers (3 females and 4 males, mean age: 25 ± 5.8 years). They were in good health, free from neurological disease, and had given informed consent to take part in the study, which was approved by the local ethics committee. The SEPs were recorded at rest with the eyes closed.

### SEP stimulation and recording parameters

The stimuli were 0.2 ms square electrical pulses delivered through a pair of Ag-AgCl electrodes cup to the left median nerve at the wrist. The intensity was adjusted for eliciting visible small thumb twitches. Random stimuli intervals (0.5 – 2 s range) were used throughout the experiment. The standard electrode positions corresponded to F3, F4, a contralateral and an ipsilateral parietal site situated 70 mm from the midline and 30 mm behind C4-C3 (these loci correspond to the site where the N20 component was maximally recorded when the contralateral wrist was stimulated [1]; all electrodes were referred to the contralateral earlobe. The on-line SEP averaging was performed using a 4-channels- NihonKohden averager (Neuropack, MEB-9100). The overall band-pass was 0.5 Hz-1.5 kHz and the analysis time was 100 ms with a sampling rate of 5 kHz. Scalp electrodes impedances were kept below 5 kΩ. Two series of 500 potentials were checked for reproducibility. After ocular artefact reduction any remaining artefact were rejected by visual inspection.

As the averager used does not permit the spectral analysis of the single sweep data and in order to independently analyze the ongoing rhythmic EEG activity from the evoked response, the raw (unaveraged) EEG data were transferred in parallel to a Pentium III personal computer with analog-to-digital converter boards (Digidata Axoscope). This analysis was only performed on the F4 channel. Off-line analysis and illustrations were then performed using the EEGLAB software [57].

### Event-related spectral perturbation (ERSP)

The EEGLAB software permits to analyze the event-related average dynamics changes in amplitude of the broad band EEG frequency spectrum and to decipher the ongoing EEG processes that may be partially time-and phase-locked to experimental events [57]. The event-related spectral perturbation measure (ERSP) may correspond to a narrow-band of event-related desynchronization (ERD) or synchronization (ERS)). Briefly, for this calculation, EEGLAB computes the power spectrum over a sliding latency window, on each epoch and normalizes each of them by its respective mean baseline spectra and then performs averaging across data trials. Each trial contains samples from -400 ms before and 400 ms after the stimulus. The size of the sliding window was of 512 data points. ERSP image provides a colour code at each image pixel indicating the reached power (in dB) at a given frequency *f *and latency *t *relative to the stimulation onset. Typically, for n trials, if *F*_*k*_*(f, t) *is the spectral estimate of trial *k *at frequency *f *and time *t*,

ERSP(f,t)=1n∑k−1n|Fk(f,t)|2
 MathType@MTEF@5@5@+=feaafiart1ev1aaatCvAUfKttLearuWrP9MDH5MBPbIqV92AaeXatLxBI9gBaebbnrfifHhDYfgasaacH8akY=wiFfYdH8Gipec8Eeeu0xXdbba9frFj0=OqFfea0dXdd9vqai=hGuQ8kuc9pgc9s8qqaq=dirpe0xb9q8qiLsFr0=vr0=vr0dc8meaabaqaciaacaGaaeqabaqabeGadaaakeaacqWGfbqrcqWGsbGucqWGtbWucqWGqbaucqGGOaakcqWGMbGzcqGGSaalcqWG0baDcqGGPaqkcqGH9aqpdaWcaaqaaiabigdaXaqaaiabd6gaUbaadaaeWbqaamaaemaabaGaemOray0aaSbaaSqaaiabdUgaRbqabaGccqGGOaakcqWGMbGzcqGGSaalcqWG0baDcqGGPaqkaiaawEa7caGLiWoaaSqaaiabdUgaRjabgkHiTiabigdaXaqaaiabd6gaUbqdcqGHris5aOWaaWbaaSqabeaacqaIYaGmaaaaaa@4D37@

To compute *F*_*k*_(*f*, *t*), EEGLAB uses the short-time Fourier transform that provides a specified time and frequency resolution.

### Inter-trial coherence (ITC)

ITC is a frequency-domain measure of the partial or exact synchronisation of activity at a particular latency and frequency to a set of experimental events to which EEG data trials are time locked. This measure corresponds to the 'phase locking factor' [58]. The term ITC refers here to its interpretation as the event-related phase coherence (ITPC), which is defined by:

ITPC(f,t)=1n∑k−1nFk(f,t)|Fk(f,t)|
 MathType@MTEF@5@5@+=feaafiart1ev1aaatCvAUfKttLearuWrP9MDH5MBPbIqV92AaeXatLxBI9gBaebbnrfifHhDYfgasaacH8akY=wiFfYdH8Gipec8Eeeu0xXdbba9frFj0=OqFfea0dXdd9vqai=hGuQ8kuc9pgc9s8qqaq=dirpe0xb9q8qiLsFr0=vr0=vr0dc8meaabaqaciaacaGaaeqabaqabeGadaaakeaacqWGjbqscqWGubavcqWGqbaucqWGdbWqcqGGOaakcqWGMbGzcqGGSaalcqWG0baDcqGGPaqkcqGH9aqpdaWcaaqaaiabigdaXaqaaiabd6gaUbaadaaeWbqaamaalaaabaGaemOray0aaSbaaSqaaiabdUgaRbqabaGccqGGOaakcqWGMbGzcqGGSaalcqWG0baDcqGGPaqkaeaadaabdaqaaiabdAeagnaaBaaaleaacqWGRbWAaeqaaOGaeiikaGIaemOzayMaeiilaWIaemiDaqNaeiykaKcacaGLhWUaayjcSdaaaaWcbaGaem4AaSMaeyOeI0IaeGymaedabaGaemOBa4ganiabggHiLdaaaa@540C@

where || represents the complex norm. The ITC measure takes values between 0 and 1. A value of 0 represents absence of synchronisation between EEG data and the time locking events; a value of 1 indicates their perfect synchronisation.

The significance levels of the ITC and ERSP were fixed at 0.001 and assessed using surrogate data by randomly shuffling the single-trial spectral estimates from different latency windows during the baseline period (bootstrap method).

### Selection of trials with pure phase resetting

The objective of the single sweep selection was to conserve only the trials for which the EEG amplitude of the filtered signal (25–35 Hz) measured around the N30 latency remained similar compared to the pre-stimulus amplitude. For this, we compared in each filtered single-sweep the root-mean-square (RMS) amplitude of the pre- and post-stimulus periods ([-200, 0 ms] and [0, +60 ms], respectively) according to the following equation:

ΔRMS=(RMSpost−RMSpre)(Max Ampl)
 MathType@MTEF@5@5@+=feaafiart1ev1aaatCvAUfKttLearuWrP9MDH5MBPbIqV92AaeXatLxBI9gBaebbnrfifHhDYfgasaacH8akY=wiFfYdH8Gipec8Eeeu0xXdbba9frFj0=OqFfea0dXdd9vqai=hGuQ8kuc9pgc9s8qqaq=dirpe0xb9q8qiLsFr0=vr0=vr0dc8meaabaqaciaacaGaaeqabaqabeGadaaakeaacqqHuoarcqWGsbGucqWGnbqtcqWGtbWucqGH9aqpdaWcaaqaamaabmaabaGaemOuaiLaemyta0Kaem4uam1aaSbaaSqaaiabdchaWjabd+gaVjabdohaZjabdsha0bqabaGccqGHsislcqWGsbGucqWGnbqtcqWGtbWudaWgaaWcbaGaemiCaaNaemOCaiNaemyzaugabeaaaOGaayjkaiaawMcaaaqaamaabmaabaGaemyta0KaemyyaeMaemiEaGNaeeiiaaIaemyqaeKaemyBa0MaemiCaaNaemiBaWgacaGLOaGaayzkaaaaaaaa@51CF@

where *Max Ampl*, the maximal amplitude of the filtered single sweep signal is measured for the pre-stimulus period ([-200, 0 ms]). Then, we selected only the trials for which the following criterion was respected:

ΔRMSstim−free¯−1SD≤ΔRMSdata≤ΔRMSstim−free¯+1SD
 MathType@MTEF@5@5@+=feaafiart1ev1aaatCvAUfKttLearuWrP9MDH5MBPbIqV92AaeXatLxBI9gBaebbnrfifHhDYfgasaacH8akY=wiFfYdH8Gipec8Eeeu0xXdbba9frFj0=OqFfea0dXdd9vqai=hGuQ8kuc9pgc9s8qqaq=dirpe0xb9q8qiLsFr0=vr0=vr0dc8meaabaqaciaacaGaaeqabaqabeGadaaakeaadaqdaaqaaiabfs5aejabdkfasjabd2eanjabdofatnaaBaaaleaacqWGZbWCcqWG0baDcqWGPbqAcqWGTbqBcqGHsislcqWGMbGzcqWGYbGCcqWGLbqzcqWGLbqzaeqaaaaakiabgkHiTiabigdaXiabdofatjabdseaejabgsMiJkabfs5aejabdkfasjabd2eanjabdofatnaaBaaaleaacqWGKbazcqWGHbqycqWG0baDcqWGHbqyaeqaaOGaeyizIm6aa0aaaeaacqqHuoarcqWGsbGucqWGnbqtcqWGtbWudaWgaaWcbaGaem4CamNaemiDaqNaemyAaKMaemyBa0MaeyOeI0IaemOzayMaemOCaiNaemyzauMaemyzaugabeaaaaGccqGHRaWkcqaIXaqmcqWGtbWucqWGebaraaa@64F6@

where Δ*RMS*_*data *_was compared to Δ*RMS*_*stim*-*free *_for two periods free of stimulus ([-350, -150 ms] and [-150, -90 ms]). After that, it was verified that the selected Δ*RMS*_*data *_distribution was comprised inside the Δ*RMS*_*stim*-*free *_distribution. This means that no significant amplitude enhancement or decrement were present in the selected trials.

### Phase histogram analysis

The degree of synchronization of the ongoing EEG oscillation was assessed by means of histograms of the instantaneous phase of the components across an ensemble of the selected trials [12]. The phase histograms of the components found in the beta/gamma (25–35 Hz) band in the selected trials were generated every 5 ms, starting 60 ms before stimulus up to 80 ms after stimulus. The Kuiper statistic *κ *coefficient was used to evaluate differences in empirical distribution functions [59] and to quantify the degree with which the phase histograms resembled a uniform density function. *κ *is defined as

κ=ln⁡〈2∑j=1∞(4j2V2−1)e−2j2V2〉,
 MathType@MTEF@5@5@+=feaafiart1ev1aaatCvAUfKttLearuWrP9MDH5MBPbIqV92AaeXatLxBI9gBaebbnrfifHhDYfgasaacH8akY=wiFfYdH8Gipec8Eeeu0xXdbba9frFj0=OqFfea0dXdd9vqai=hGuQ8kuc9pgc9s8qqaq=dirpe0xb9q8qiLsFr0=vr0=vr0dc8meaabaqaciaacaGaaeqabaqabeGadaaakeaaiiGacqWF6oWAcqGH9aqpcyGGSbaBcqGGUbGBdaaadaqaaiabikdaYmaaqahabaWaaeWaaeaacqaI0aancqWGQbGAdaahaaWcbeqaaiabikdaYaaakiabdAfawnaaCaaaleqabaGaeGOmaidaaOGaeyOeI0IaeGymaedacaGLOaGaayzkaaGaemyzau2aaWbaaSqabeaacqGHsislcqaIYaGmcqWGQbGAdaahaaadbeqaaiabikdaYaaaliabdAfawnaaCaaameqabaGaeGOmaidaaaaaaSqaaiabdQgaQjabg2da9iabigdaXaqaaiabg6HiLcqdcqGHris5aaGccaGLPmIaayPkJaGaeiilaWcaaa@4E5F@

with

V=(N+0.155+0.24N).max⁡i=1,...,L[SN(xi)−F0(xi)]+max⁡i=1,...,L[F0(xi)−SN(xi)],
 MathType@MTEF@5@5@+=feaafiart1ev1aaatCvAUfKttLearuWrP9MDH5MBPbIqV92AaeXatLxBI9gBaebbnrfifHhDYfgasaacH8akY=wiFfYdH8Gipec8Eeeu0xXdbba9frFj0=OqFfea0dXdd9vqai=hGuQ8kuc9pgc9s8qqaq=dirpe0xb9q8qiLsFr0=vr0=vr0dc8meaabaqaciaacaGaaeqabaqabeGadaaakeaacqWGwbGvcqGH9aqpdaqadaqaamaakaaabaGaemOta4ealeqaaOGaey4kaSIaeGimaaJaeiOla4IaeGymaeJaeGynauJaeGynauJaey4kaSYaaSaaaeaacqaIWaamcqGGUaGlcqaIYaGmcqaI0aanaeaadaGcaaqaaiabd6eaobWcbeaaaaaakiaawIcacaGLPaaacqGGUaGldaWfqaqaaiGbc2gaTjabcggaHjabcIha4bWcbaGaemyAaKMaeyypa0JaeGymaeJaeiilaWIaeiOla4IaeiOla4IaeiOla4IaeiilaWIaemitaWeabeaakmaadmaabaGaem4uam1aaSbaaSqaaiabd6eaobqabaGccqGGOaakcqWG4baEdaWgaaWcbaGaemyAaKgabeaakiabcMcaPiabgkHiTiabdAeagnaaBaaaleaacqaIWaamaeqaaOGaeiikaGIaemiEaG3aaSbaaSqaaiabdMgaPbqabaGccqGGPaqkaiaawUfacaGLDbaacqGHRaWkdaWfqaqaaiGbc2gaTjabcggaHjabcIha4bWcbaGaemyAaKMaeyypa0JaeGymaeJaeiilaWIaeiOla4IaeiOla4IaeiOla4IaeiilaWIaemitaWeabeaakmaadmaabaGaemOray0aaSbaaSqaaiabicdaWaqabaGccqGGOaakcqWG4baEdaWgaaWcbaGaemyAaKgabeaakiabcMcaPiabgkHiTiabdofatnaaBaaaleaacqWGobGtaeqaaOGaeiikaGIaemiEaG3aaSbaaSqaaiabdMgaPbqabaGccqGGPaqkaiaawUfacaGLDbaacqGGSaalaaa@7CAC@

where *F*_*0*_*(x*_*i*_*) *and *S*_*N*_*(x*_*i*_*) *are the mean pre-stimulus reference period [-60 ms, -5 ms] and the actually observed cumulative distribution function, respectively; *N *is the effective number of data points, *L *the number of bins in the histrogram and *x*_*i *_the upper bound of bin *i*.

The more the observed *S*_*N*_*(x*_*i*_*) *is different from the reference *F*_*0*_*(x*_*i*_*)*, the more *κ *will be negative. In order to provide more evidence to demonstrate the reliability of the effect across subjects a Z-score of *κ *is given.

Data were analyzed using a one-way ANOVA test and Bonferroni's post-hoc test after assessing their normality by a Kolmogorov-Smirnov test (Statistica 7.1, Statsoft). Differences were considered significant at *P *< 0.05. Results are expressed as means ± SD.

## Authors' contributions

GC conceived the study and wrote a first draft of the manuscript, AMC performed the majority of the experiments, CD performed statistical analysis, AMC, CD, AB, FL, AL, BD participated in analysis of the data. All the authors participated in the interpretation of the data. GC, AMC and BD wrote the final manuscript. All authors read and approved the final manuscript.
